# Integrating patients in time series clinical transcriptomics data

**DOI:** 10.1093/bioinformatics/btae241

**Published:** 2024-06-28

**Authors:** Euxhen Hasanaj, Sachin Mathur, Ziv Bar-Joseph

**Affiliations:** Machine Learning Department, Carnegie Mellon University, Pittsburgh, PA 15213, United States; R&D Data and Computational Sciences, Sanofi, Cambridge, MA 02141, United States; Machine Learning Department, Carnegie Mellon University, Pittsburgh, PA 15213, United States; R&D Data and Computational Sciences, Sanofi, Cambridge, MA 02141, United States; Computational Biology Department, Carnegie Mellon University, Pittsburgh, PA 15213, United States

## Abstract

**Motivation:**

Analysis of time series transcriptomics data from clinical trials is challenging. Such studies usually profile very few time points from several individuals with varying response patterns and dynamics. Current methods for these datasets are mainly based on linear, global orderings using visit times which do not account for the varying response rates and subgroups within a patient cohort.

**Results:**

We developed a new method that utilizes multi-commodity flow algorithms for trajectory inference in large scale clinical studies. Recovered trajectories satisfy individual-based timing restrictions while integrating data from multiple patients. Testing the method on multiple drug datasets demonstrated an improved performance compared to prior approaches suggested for this task, while identifying novel disease subtypes that correspond to heterogeneous patient response patterns.

**Availability and implementation:**

The source code and instructions to download the data have been deposited on GitHub at https://github.com/euxhenh/Truffle.

## 1 Introduction

Transcriptomics data has been collected and profiled in clinical and drug response studies for over a decade (Meyer *et al.* 2013). In most cases, researchers profile bulk expression, though more recently single-cell data was also profiled in such studies (Wang *et al.* 2020). The main goal of these studies is to reconstruct networks and systems that are activated in response to the disease, drug, or vaccine, over time (Almon *et al.* 2003, Huang *et al.* 2009).

A major challenge in the analysis of data from clinical trials is the fact that different individuals may display different response *dynamics* (Bar-Joseph *et al.* 2012, Ding *et al.* 2022). Even if the same biological process is activated, based on baseline differences (related to age, gender, prior disease history, etc.), these individuals may respond faster or slower to the same treatment. Furthermore, same-day visits do not correspond to the same disease state which makes it challenging to rely on the measured time points for integrating data across these patients. Another challenge is the heterogeneous responses from different individuals. While a single response trajectory is possible, often we observe a (small) number of endotypes. “Endotypes” are subtypes of a disease characterized by different pathogenic mechanisms (Lötvall *et al.* 2011, Czarnowicki *et al.* 2019, Battaglia *et al.* 2020) which can have an impact on the specific optimal treatment. Each of the endotype groups may respond differently to the same treatment and so the overall set of patients cannot be directly integrated when studying treatment or vaccine response.

Several methods have been developed to address the first challenge (aligning patients) (Listgarten *et al.* 2004, Lin *et al.* 2008). These often use expectation-maximization (EM) like methods. In these approaches, genes are represented as continuous curves and individuals are assigned to different time points along these (Bar-Joseph *et al.* 2003). Such methods have been widely applied (Behnke *et al.* 2010, Czarnewski *et al.* 2019) but they still suffer from several drawbacks. First, the continuous expression assumption may be problematic when sampling rates are sparse (genes can change a lot between two consecutive measurements) and second, they cannot reconstruct trajectories for multiple subsets of patients but rather assume a homogeneous response among all patients.

Another direction that was explored, especially in the single-cell space, is that of trajectory inference. Unlike the EM methods, these approaches assume the presence of multiple states in the data and allow for multiple subsets or branching. These methods range from linear or tree-based, to more recent adaptations of RNA velocity (Saelens *et al.* 2019, Lange *et al.* 2022). However, most of these methods assume no relationships between cells or samples. Only a few methods have focused on the case when samples come from different time points as is often the case with clinical trials data. For example, Tempora (Tran and Bader 2020) assigns temporal scores to each cluster of cells which are used to determine the direction of the edges. Psupertime fits a series of ordinal logistic regression models that separate time points while trying to find a small number of genes that influence the resulting order (Macnair *et al.* 2022). However, these single-cell methods assume a very large number of samples (in the thousands or tens of thousands) which is not available for most clinical studies including the ones analyzed in this paper. In addition, they usually do not explicitly map the different subgroups within the data, leaving it for subsequent, post-processing, analysis.

In this work, we present Trajectory Inference via Multi-commodity Flow with Node Constraints (Truffle), a method that performs pseudotime ordering of samples in short time series data. Truffle is based on the multi-commodity flow algorithm (Leighton *et al.* 1995) which generalizes minimum cost flow problems to include multiple source and sink nodes. Each sample in our data can be seen as either a source or a sink node and we are interested in recovering directed paths between these that minimize a cost function (typically some distance in gene space). The advantage of Truffle is that these trajectories can be constrained to satisfy timing restrictions and to pass through other nodes which correspond to intermediate disease states not present in the patient specific time series. Endotypes are then determined by constructing a state diagram for different subsets of patients. Truffle allows for the possibility of recovering contrasting endotypes since trajectories are inferred for each patient rather than for the entire dataset.

We tested Truffle on several microarray and bulk RNA-seq datasets. As we show, Truffle can accurately identify relevant disease trajectories and pathways, improving upon prior methods for clinical time series data and methods for single-cell data. A number of novel trajectories identified by Truffle suggest new subsets of patients that can benefit from precision medicine.

## 2 Materials and methods

### 2.1 Data and preprocessing

We used three public time series datasets with the following GEO accession numbers GSE171012 (psoriasis), GSE212041 (COVID-19), and GSE112366 (Crohn’s disease) (VanDussen *et al.* 2018, LaSalle *et al.* 2022, Liu *et al.* 2022) (Table 1).

**Table 1.** Clinical data used in this study.^a^

Disease	Number of				Metadata		
	Samples	Genes	Patients${\;}^{+(-)}$	Visits	Time points	Tissue	Treatment
Crohn’s	231	11,133	108 (26)	3	WK0, WK8, WK44	Ileum	ustekinumab
COVID-19	650	33,142	304 (8)	3	D0, D3, D7	Blood	N/A
Psoriasis	55	16,369	15 (11)	4	Pre^b^, WK2, WK4, WK12	Lesion	secukinumab

a All three datasets contain missing values. We show both the number of patients who tested positive (+) and the number of healthy control patients (−).

b Pretreatment week.

Raw gene counts were downloaded from NCBI GEO for the two RNA-seq datasets (psoriasis and COVID-19). Only protein-coding genes that had >0.25 counts per million (CPM) in at least 1% of the samples were kept. In the case of duplicated gene identifiers, the gene with the highest mean expression was considered. Datasets were then normalized for their guanine-cytosine (GC) content and trimmed mean of M-values (TMM) was performed (Robinson and Oshlack 2010). If batch information was present, ComBat was used to extract batch-corrected expression values (Johnson *et al.* 2006). Only samples with disease/treatment were used for pseudo-ordering. For microarray data, in the case of multiple probesets belonging to a protein-coding gene, only the one with the highest expression was kept. The Crohn’s dataset was pre-normalized by Robust Multichip Analysis (RMA).

We removed symptomatic ${\text{COVID}-19}^{-}$ from the COVID-19 data and kept only the patients who tested positive for the disease.

### 2.2 Assignment of disease states through clustering

To obtain disease states, we clustered the samples. We followed a standard practice that is also adopted by other computational tools such as Seurat (Hao *et al.* 2024). We first ran principal component analysis (PCA) to obtain low dimensional embedding vectors which were then used to construct a fuzzy simplicial set as done by Uniform Manifold Approximation and Projection (UMAP) (McInnes *et al.* 2018). We adjusted the number of neighbors based on the total number of samples—using 15 for Crohn’s, 20 for COVID-19, and 5 for psoriasis. Larger numbers resulted in highly connected graphs. This connectivity graph is the input for both Leiden clustering (Traag *et al.* 2019), and multi-commodity flow (below).

To assign states to biological processes, we performed gene set enrichment analysis (GSEA) (Subramanian *et al.* 2005) using the prerank function of GSEApy (Fang *et al.* 2023). Genes were ranked based on the following score:

$$\text{gene}\;{\text{score}}_{i}=-{\;\text{log}\;}_{10}(\text{adj}.\;p\text{-value})\cdot {\;\text{log}\;}_{2}(\text{FC})$$

where in the first term, adjusted *p* values were obtained from a two-sided Kolmogorov-Smirnov test (Massey 1951) comparing the diseased and healthy sets of patients, and the second term is the log fold-change in gene expression between the two sets. We rely on the gene ontology (GO) biological processes marker set for the enrichment analysis in this work (Ashburner *et al.* 2000).

### 2.3 Multi-commodity flow with node capacity constraints

The multi-commodity flow problem with node capacity constraints is defined as follows. Consider a directed graph G=(V,E), where an edge (u,v)∈E has an associated cost $c_{u,v}$. We are given a set of *K* commodities K:=[K]. The $i^{\text{th}}$ commodity is defined by a source and sink node $\left(s_{i},t_{i}\right)$.

Multi-commodity flow can be used to model patient trajectories. Assume for simplicity patients with only two visits each. In this setup, each patient corresponds to one commodity, and the two visits represent its source *s* and sink *t*. The objective is to recover a smooth disease trajectory between these two endpoints. If the data contains patients with diverse disease states, we can assume that some of the samples will lie “in between” *s* and *t*. The shortest path between these two nodes in the neighbors graph captures this smooth transition. By setting edge and node capacities we force the algorithm to look for robust paths (defined here as paths with similar state transitions even though they share no edges). Finally, if a patient has more than two time points, we consider each transition separately. For example, a time series a→b→c is split into two commodities a→b and b→c.

Specifically to use multi-commodity for trajectory inference, we use the following constrains. For every commodity *i*, we wish to learn separate functions $f_{i}:E\to \{0,1\}$ that satisfy the following constraints:

1. **Max edge capacity**: the total amount of commodity that passes over an edge does not exceed its capacity
  $$\forall (u,v)\in E:\sum_{i\in K}\;f_{i}(u,v)\leq C.$$
2. **Flow conservation**: flow must fully exit source nodes and enter sink nodes. For all i∈K:
  $$\begin{array}{c} \forall n\in V:\sum_{w\in V}\;f_{i}(n,w)\;-\;f_{i}(w,n)=\{\begin{array}{cc} 1 & \text{if}\;n\;\text{is}\;\text{the}\;i^{\text{th}}\;\text{source}\\ -1 & \text{if}\;n\;\text{is}\;\text{the}\;i^{\text{th}}\;\text{sink}\\ 0 & \text{otherwise} \end{array} \end{array}$$
  Given a node capacity N>0, we also consider the following constraint:
3. **Max node capacity**: the total amount of commodity that passes through a node does not exceed its capacity
  $$\forall w\in V:\sum_{i\in K}\;\sum_{u\in V,u\neq w}\;f_{i}(u,w)\;\leq \;N.$$

**Figure 1.**
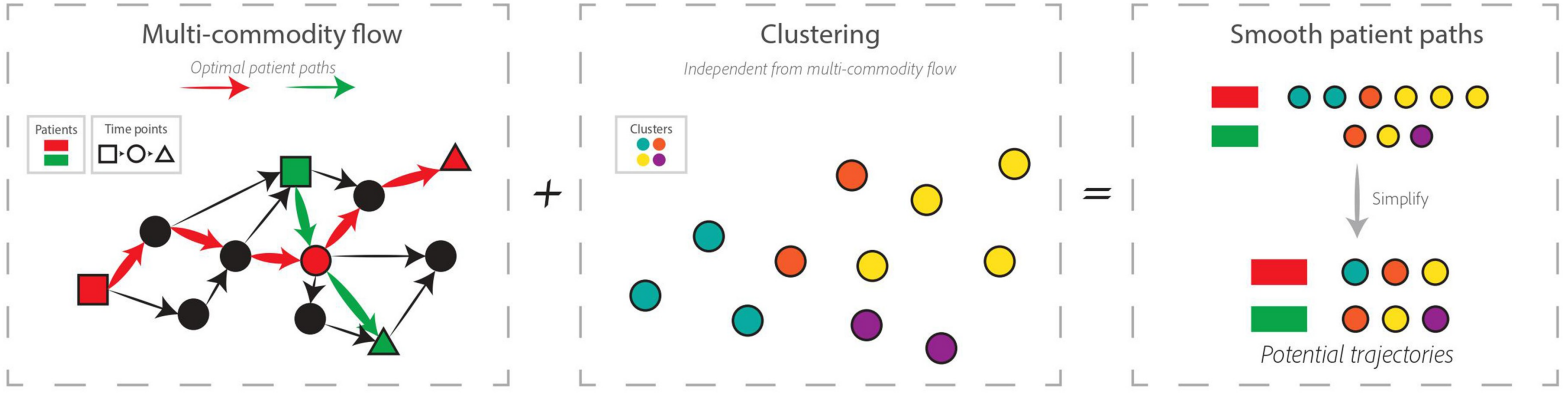
Schematic illustration of Truffle. For each patient, our flow algorithm returns a trajectory that passes through intermediate nodes for a smoother response. These trajectories are then aligned with the clustering results to obtain a state diagram. Finally, by estimating state initial and final probabilities from the data, we can compute and study the top directed trajectories.

Along with flow conservation, constraint three guarantees limits on both incoming and outgoing flow. This variant of multi-commodity flow with node capacity constraints has also been explored before (Charikar *et al.* 2019). The integer problem has been shown to be NP-complete (Even *et al.* 1975), however, its fractional form (setting the codomain of *f* to be *[*0, 1*]*) can be solved in polynomial time through linear programming. We use the open source Python optimization library pyomo (Bynum *et al.* 2021) and the glpk solver (Oki 2012). It is worth noting that faster commercial solvers exist (Meindl and Templ 2013) (Fig. 1).

In the general formulation of the problem, each commodity can have a demand *D*, and each edge can have a capacity *C* (Leighton *et al.* 1995). Since a priori we do not have any preference for individuals, we set D=1 for all commodities. We set C=1 for psoriasis and Crohn’s datasets. For the COVID-19 data, the problem was infeasible for C=1, so we used C=2. Enforcing edge and node capacities prevents outliers and errors in the data from having a large impact. An example has been provided in Supplementary Fig. S1.

### 2.4 Obtaining flow satisfying solutions

We learn *f* by optimizing the following target function

$$U=\sum_{(u,v)\in E}\;\left(c_{u,v}\sum_{i\in K}\;f_{i}(u,v)\right)$$

Recall that $c_{u,v}$ is a cost function. As we are concerned with smooth trajectories, this is initialized as the Euclidean distance between the PCA embeddings for nodes *u* and *v*.

Note that for any given commodity defined by source $s_{i}$ and target $t_{i}$, most of the edges “far away” from $s_{i}$ and $t_{i}$ will not be picked by the solver. We can incorporate this observation into our problem by considering only edges that belong to any path $s_{i}\to t_{i}$ of length ≤ℓ for some ℓ. This reduces the runtime for large datasets without compromising the optimality of the solution. For the smaller datasets, we found that the solution to this modified problem was similar to the original one. For the COVID-19 data, we set ℓ=4. Unreachable commodities were removed (17%).

### 2.5 Trajectory inference from optimal flow paths

After obtaining a path for each patient, we aggregate this information in the form of a state-transition matrix. In this work, we estimate initial and final state probabilities from the data, although domain expertise or priors determined from larger knowledge bases can be also used. Finally, we can then compute the most likely trajectories by performing random walks of a desired length. This is preferred over simply counting the occurrence of each path since in that case we could miss trajectories which are not identical, but show the same trend. For example, the paths 0−5−2−7 and 0−5−3−2−7 are different, but likely correspond to a similar disease trajectory. Our setup would assign a high probability to transitions 0−5 and 2−7.

### 2.6 STEM analysis of learned trajectories

To determine groups of genes that follow similar transcriptional programs, we perform Short Time-series Expression Miner (STEM) analysis (Ernst and Bar-Joseph 2006). We performed STEM normalization on gene expression values and used the default number of profiles (50), except for paths of length 2 where the maximum possible number is 16. Larger values for the number of profiles resulted in many redundant profiles that were nearly identical. For psupertime only, we reduced the “Minimum Absolute Expression Change” to 0, since psupertime normalized expression values were in a much smaller range than for the other two methods.

## 3 Results

We developed a method to perform pseudotime ordering of multiple short times series clinical data based on optimal flow algorithms (Fig. 1). Our method takes as input gene expression data from multiple subjects along with their specific time point, and tries to reconstruct trajectories that describe distinct disease endotypes. As a proof of concept, we first performed a simulation study with randomly generated data. Truffle accurately recovered the simulated trajectories in this study (Supplementary Fig. S2). To further validate our method, we used clinical data for psoriasis, COVID-19, and Crohn’s disease (Table 1). We compare our method against prior work developed for similar tasks including Tempora, psupertime, as well as a baseline that assigns endotypes based solely on clustering analysis. The set GO Biological Processes was used for Tempora.

### 3.1 Truffle recovers trajectories that indicate regeneration and reduction of inflammation in patients with psoriasis

**Figure 2.**
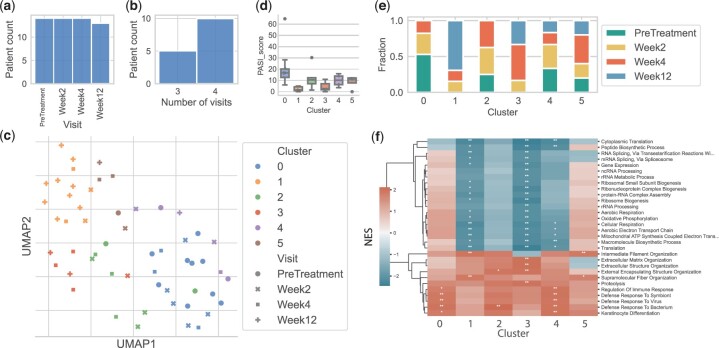
Clustering analysis of the psoriasis dataset. (a) and (b) Distribution of visits across patients. (c) UMAP plot of cluster assignments. (d) Boxplots of PASI scores for each cluster. (e) Relative frequency of visits by cluster. (f) Top GO terms for each cluster against healthy samples. We used a KS test to rank the genes. A (∗) symbol means the category was statistically significant [(∗∗)≡q≈0 and (∗)≡q ≤ 0.05].

We tested Truffle on bulk RNA data from psoriasis patients treated with secukinumab. The data spans 12 weeks and most patients have data for all four time points (Fig. 2a and b). Leiden clustering identified six states (Fig. 2c). Cluster 0 predominantly consists of pre-treatment samples (50%) and contains no samples from week 12. Judging by the PASI scores (Fig. 2d), this cluster represents severe chronic plaque psoriasis. GO analysis shows significant upregulation of genes involved in the regulation of immune response (FDR ≤0.001) and defense response to virus & bacterium (FDR ≈0, Fig. 2f) when compared to healthy samples. We also see significant upregulation for keratinocyte differentiation (FDR ≤0.001) which is a hallmark of moderate-severe disease states (Ma *et al.* 2023). Other immune-related processes such as Neutrophil Chemotaxis, Antimicrobial Humoral Response, and Regulation Of Interferon-Beta Production were also up-regulated in this cluster (Supplementary Fig. S3d). In contrast, for cluster 1, approximately 70% of the samples are from week 12 and there are no samples assigned to this cluster from the pre-treatment week. The PASI scores for cluster 1 were also the lowest among all clusters (an average of 2.3). This cluster is enriched for intermediate filament and supramolecular structure organization, and keratinocyte differentiation is no longer significant. Downregulation of processes related to regulation of gene expression is also seen as a result of drug action, along with a reduced immune response.

**Figure 3.**
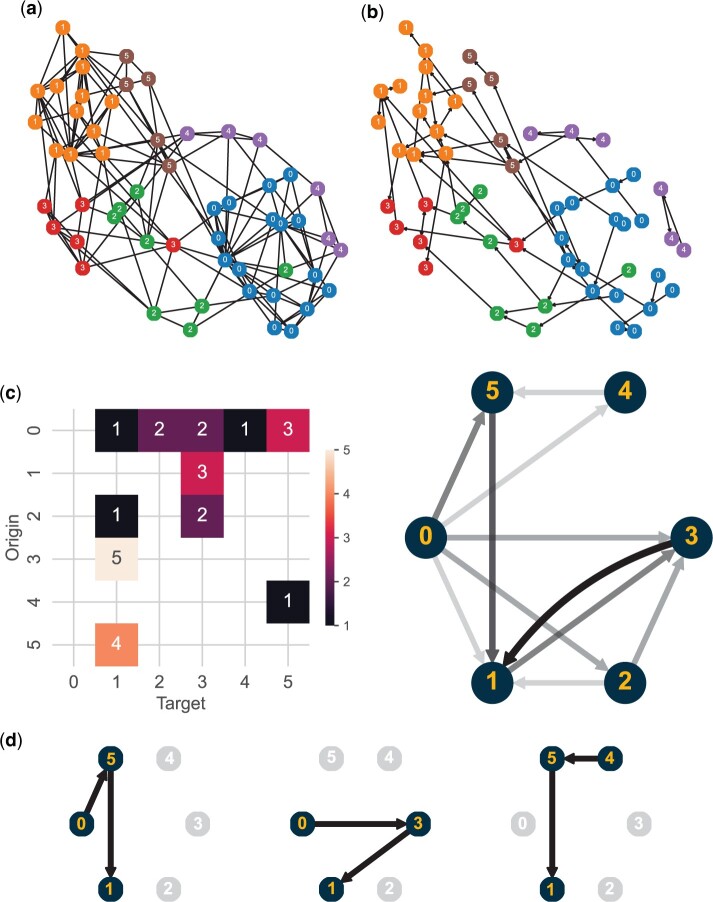
Truffle state diagram and top trajectories for the psoriasis dataset. (a) Original connectivity graph obtained using fuzzy simplicial sets and (b) the graph corresponding to all the low-cost trajectories selected by Truffle (right). We used an edge capacity of 1 and a node capacity of 3 for this dataset. (c) The pruned state diagram describing the main state transitions in the Truffle network. Repeated states were collapsed into one, hence, no self-loops are shown. (d) The top paths identified by Truffle.

**Figure 4.**
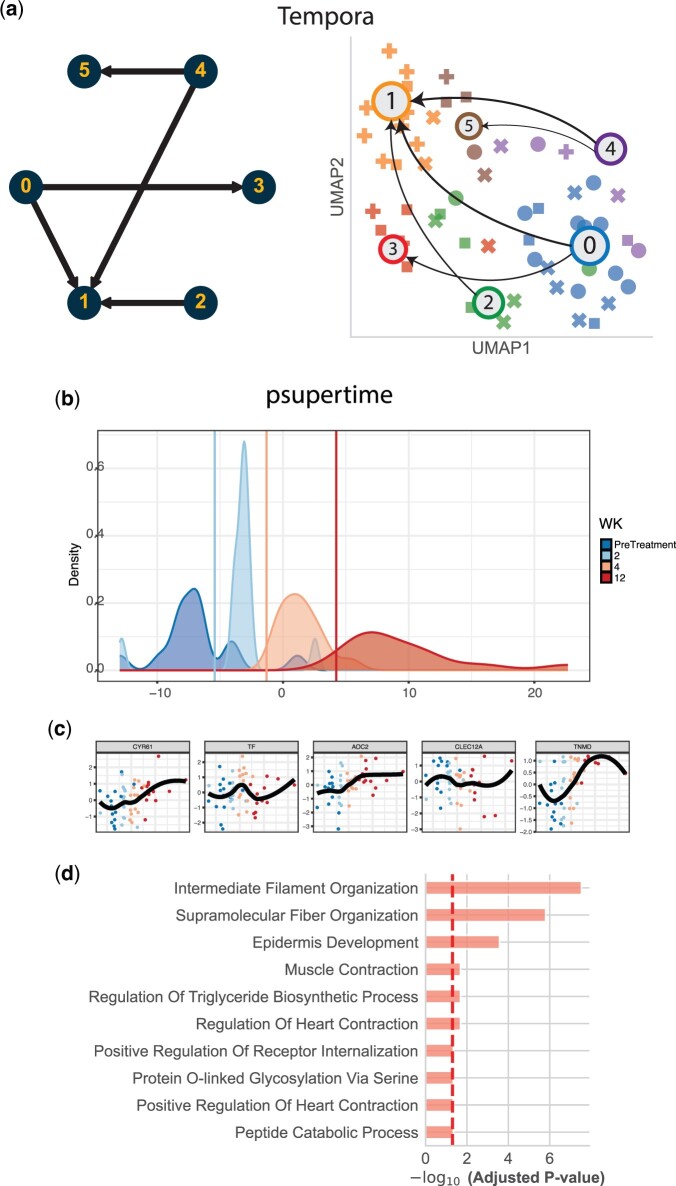
Trajectories uncovered by Tempora and psupertime for the psoriasis dataset. (a) Transition graph identified by Tempora. Five trajectories of length 1 were identified. (b) Separation of time points by psupertime. The *y* axis is the density of each time point and the *x* axis is the temporal ordering. (c) The top five genes identified as relevant by psupertime. These correspond to the genes with the largest absolute coefficients. (d) The top GO terms for all the relevant genes (294). Subfigures (b) and (c) were generated using psupertime.

We first looked at the most common cluster transitions using patients’ samples timeline without cost constraints. We found that three patients transitioned from state 0→1, and two remained at state 4. All the remaining transitions were exclusive to only one patient. Next, we ran Truffle to uncover smoother response trajectories. Figure 3 shows the state diagram identified by Truffle as well as the top three paths. The transition 0→1 was supplemented with two intermediate states, 5 and 3. GO analysis (Fig. 2f) shows that state 5 is characterized by a downregulation of defense response mechanisms when compared to state 0, while serving as an intermediary for a number of downregulated terms in state 1. On the other hand, state 3 is characterized by an upregulation of extracellular matrix organization which plays a role in tissue regeneration. Among the baselines, Tempora was able to recover paths of length 1 only (Fig. 4a). However, it correctly identified state 1 as a terminal state, but also 3 and 5. Psupertime identified 294 genes which vary coherently with time. GO analysis shows that these genes are enriched for intermediate filament and supramolecular fiber organization, as well as epidermis development. However, no significant terms involving defense response were found for the psupertime results.

**Figure 5.**
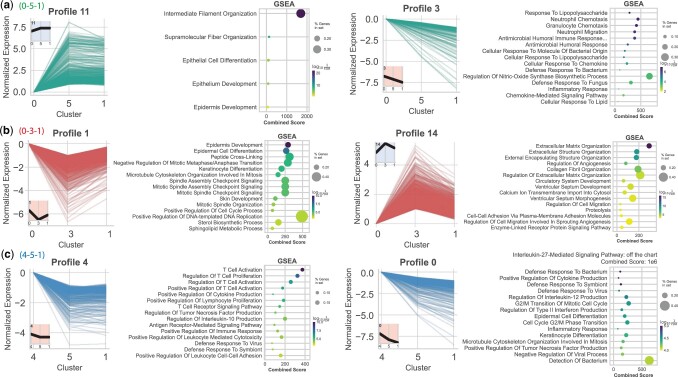
Selected STEM profiles for the top three Truffle trajectories in the psoriasis dataset. (a–c) Two selected profiles for each of the three trajectories. In (c, right) “IL-27 Mediated Signaling Pathway” obtained a very high combined score (1e6), hence, was removed from the plot for clarity. The full list of profiles can be found in the supplement.

Finally, we performed STEM analysis on the top three trajectories identified by Truffle. Profiles involving upregulation of epidermis development and downregulation of defense response overlapped across all three trajectories. Trajectories 0−5−1 and 4−5−1 contained decreasing profiles which were significantly enriched for genes involved in “IL-27-Mediated Signaling Pathway” [Combined Score ≥1e6, Fig. 5c (right) and Supplementary Fig. S3c]. These two trajectories differ in their initial state only. While states 0 and 4 are both enriched for defense response, state 4 shows a downregulation of terms such as cytoplasmic translation and other biosynthetic processes.

### 3.2 Truffle identifies different immune responses to COVID-19

**Figure 6.**
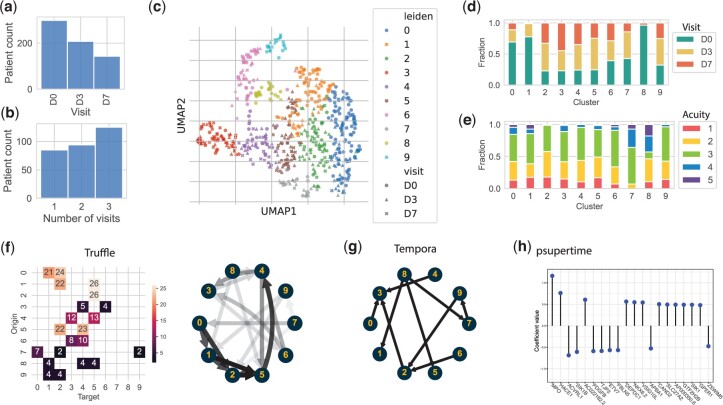
Clustering and trajectory analysis for the COVID-19 dataset. (a) and (b) Distribution of visits and distribution of visit counts per patient. (c) Clustering 650 samples from 304 patients. (d) Relative frequency of visits and (e) acuity scores per cluster. (f) A pruned diagram of top state transitions identified by Truffle. Pruning was performed by taking the fewest top edges that amount to ≥50% of a node’s outgoing weight. (g) The tree learned by Tempora. Final states are 3, 1, and 5. (h) The top genes that vary with time according to psupertime (plot obtained from psupertime).

We repeated the analysis with samples from a larger dataset of COVID-19 patients collected at days 0, 3, and 7. Clustering analysis identified 10 states (Fig. 6c). State 8 consisted of day 0 samples, and showed the highest acuity scores (Fig. 6d and e). State 0 showed significant upregulation of inflammatory response and other defense mechanisms when compared to healthy samples (FDR ≈0, Supplementary Fig. S5). State 1 was similarly enriched for “Defense Response to Virus,” but not for inflammation. About 20% of all patients ended in state 2, which differed from healthy samples only in it being significantly enriched for Antimicrobial Humoral Response and Defense Response To Bacterium (FDR ≈0). This suggests that this is a milder state than the previous two, also confirmed by acuity scores where cluster 2 is the only one containing no samples with acuity 4 or 5 (Fig. 6e). Across all three time points, most patients (10) moved from state 0 to state 2. This was also the top trajectory captured by Truffle (factoring in initial and terminal probabilities for each state, Fig. 6f). In contrast, this trajectory was not recovered by Tempora (Fig. 6g).

Next, we studied the top trajectories identified by Truffle at varying levels of resolution. The top trajectories of lengths 3 and 4 were $T_{1}:=0-1-5-2$, $T_{2}:=0-1-5-4$, and $T_{3}:=0-1-2-5-4$, $T_{4}:=0-2-5-4-3$, respectively. For brevity, since $T_{2}$ is a subsequence of $T_{3}$, we only look at $T_{3}$, although $T_{2}$ could be an endotype in its own right describing a “faster” response.

STEM analysis of $T_{1}$ assigned >4000 genes to profile 49 (Supplementary Fig. S4a). GO analysis showed that ∼50 genes in profile 49 were involved in sensory perception of smell (FDR =0.02), a common symptom of COVID-19 (Parma *et al.* 2020). We see an upregulation of these genes from 0→5, but a downregulation from 5→2.

On the other end, for $T_{3}$, STEM assigned >9000 genes to a strictly increasing profile (profile 41, Supplementary Fig. S4a). This profile was also enriched for processes related to sensory perception of smell, but this time we see an upregulation of related genes across all four temporal steps. Profile 2 ($T_{3}$) and profile 9 ($T_{4}$) indicate downregulation of immune response. Profile 9 is gradual. Looking at GO enrichment of the final state of $T_{4}$ (cluster 3), we observe a return to baseline (healthy) for various defense response processes and downregulation of gene regulation activities (Supplementary Fig. S5).

Tempora, on the other hand, identified only two paths of length ≥2. These were $Q_{1}:=6-2-3$ and $Q_{2}:=8-7-9-2-3$. Three significant STEM profiles were determined for $Q_{1}$, none of which was significantly enriched for any GO process (FDR =0.05). For $Q_{2}$, STEM returned 11 significant profiles. Among these, only three were enriched for GO processes (Supplementary Fig. S4b). Profile 10 was enriched for sensory perception of smell, and profile 7 was enriched for the only term “Positive Regulation of NF-kappaB Transcription Factor Activity.” Meanwhile, profile 37 showed an initial increment, followed by a monotone decrement of processes related to signaling. Finally, psupertime identified 462 relevant genes. GO analysis using these genes returned only one process: “Hydrogen Peroxide Catabolic Process” (FDR =0.007).

### 3.3 Truffle identifies two contrasting response mechanisms to ustekinumab in patients with Crohn’s disease

Finally, we tested Truffle on microarray data from patients with Crohn’s disease treated with ustekinumab (VanDussen *et al.* 2018). The data was collected at weeks 0, 8, and 44. Clustering analysis revealed eight distinct states. States 1 and 4 were not statistically different from healthy samples. States 0, 3, and 6 expressed genes enriched for inflammatory response, while cluster 2 showed a downregulation of the process (Supplementary Fig. S6b and c).

The top Truffle trajectories of length 2 were $C_{1}:=3-4-1$ and $C_{2}:=2-5-0$. $C_{1}$ transitions from a state with inflammation into two healthy states, suggesting that patients along this path saw improvement from the drug. In contrast, for $C_{2}$ we see an activation of immune response in its final state (cluster 0). Indeed, about 14 patients were clustered under state 0 at week 44, suggesting that they showed partial response to the drug. STEM analysis of $C_{1}$ returned several decreasing profiles which were enriched for inflammatory response. In contrast, $C_{2}$ was assigned increasing profiles enriched for immune response and activation of T cells (Supplementary Fig. S6d). Thus, Truffle was able to recover two contrasting endotypes for patients in this study.

## 4 Discussion

Several trajectory inference methods have been developed to date and these differ in representation power and assumptions made (Saelens *et al.* 2019). Most of the work has focused on single cell with much less focus on data collected in clinical studies. Here we focus on studies that profile a small number of time-points in multiple patients. To analyze such data, we developed Truffle which respects the time ordering of samples for a given patient, and obtains patient journeys through the disease/treatment process. Truffle is based on multi-commodity flow by splitting short time series into source and target nodes. These are then connected through a path that travels through other intermediate nodes in order to generate a smooth path. We tested Truffle on several time series datasets and compared it to two other methods developed for similar tasks.

For the psoriasis dataset, all patients display a significant health improvement after treatment with secukinumab as indicated by their PASI scores and GO analysis of the terminal state. Since patients respond differently to the treatment, we sought to understand different endotypes within the patient population. Clustering analysis does not lead to accurate grouping of disease subtypes. Some of the other methods were able to capture the improvement either by identifying a healthy final state (Truffle, Tempora) or by showing enrichment for healing biological processes (psupertime). However, Tempora identified only paths of length 1, thus providing lower resolution into the drug response progression, while psupertime does not provide details into different response mechanisms or endotypes due to its linearity assumption. Only Truffle was able to capture temporal dynamics of the treatment process among different patients and obtain different endotypes. For example, Truffle recovered two paths which end in a healthy state but travel through different states. Both show the downregulation of *IL-27* and its pathway genes. Reduction in expression of type I & II interferons (IFNs) and/or tumor necrosis family (TNF) receptors, which are regulators of *IL-27*, has been previously observed as part of the recovery (Povroznik and Robinson 2020). Furthermore, *IL-27* was previously reported to promote the onset of psoriasis (Shibata *et al.* 2010). However, they also differ in other pathways. One of these trajectories was characterized by an upregulation of extracellular matrix organization (ECM) and downregulation of intermediate filament organization (IFO), while for the other trajectory we observed the opposite. Prior work has shown that activation of ECM is related to the severity of psoriasis (Wagner *et al.* 2021). We hypothesize that the upregulation of ECM may be an intermediary stage of slow responders. Results show that a subset of patients quickly attained normalization of keratinocyte differentiation (Figs 2 and 3 clusters 1, 3, 5). Such patients can be deemed as super/fast responders to therapy. These patients can be further investigated to better tailor personalized therapy.

For the COVID-19 dataset, prior methods failed to recover smooth trajectories with any significant GO terms. Tempora recovered trajectories that oscillate between time points, which makes them hard to interpret, and psupertime returned only one significant GO process, likely because this linear method was forced to combine heterogeneous subtypes in its trajectories. Truffle identified several trajectories, including ones which showed a downregulation of defense response over time and others where this response was reinstated at day 7. This was confirmed by a reduction of sensory perception of smell during this time step.

While the applications we presented are mainly focused on immunology, we believe that Truffle can also be applied to oncology time series data and that it can also be integrated with time series data from other sources including electronic health records or claims databases.

While successful, Truffle has a few limitations. The datasets we used in this study contained at most 650 samples. The open-source linear solver we used to optimize a graph of this size may not scale to graphs with several thousands of samples. In this case, several simplifications to the problem may need to be introduced, such as limiting the set of edges a commodity can be transported over. For the specific datasets we evaluated, Truffle took 0.12 s to run for the small psoriasis dataset and 22 s for the larger COVID-19 dataset (ℓ=4) (tests performed on a MacBook Pro with an M3 Pro Max chip). In addition, faster commercial solvers can also be used.

To conclude, Truffle is a method for integrating patient data in time series transcriptomics studies. It is able to both, identify patient trajectories and subgroups within a population. Truffle is available as an open-source software from the link in the abstract.

## Supplementary Material

btae241_Supplementary_Data

## References

[btae241-B1] Almon RR , DuBois DC, Pearson KE et al Gene arrays and temporal patterns of drug response: corticosteroid effects on rat liver. Funct Integr Genomics 2003;3:171–9. 12928814 10.1007/s10142-003-0090-x PMC4207265

[btae241-B2] Ashburner M , Ball CA, Blake JA et al Gene ontology: tool for the unification of biology. The Gene Ontology Consortium. Nat Genet 2000;25:25–9. 10802651 10.1038/75556 PMC3037419

[btae241-B3] Bar-Joseph Z , Gerber GK, Gifford DK et al Continuous representations of time-series gene expression data. J Comput Biol 2003;10:341–56. 12935332 10.1089/10665270360688057

[btae241-B4] Bar-Joseph Z , Gitter A, Simon I. Studying and modelling dynamic biological processes using time-series gene expression data. Nat Rev Genet 2012;13:552–64. 22805708 10.1038/nrg3244

[btae241-B5] Battaglia M , Ahmed S, Anderson MS et al Introducing the endotype concept to address the challenge of disease heterogeneity in type 1 diabetes. Diabetes Care 2020;43:5–12. 31753960 10.2337/dc19-0880 PMC6925574

[btae241-B6] Behnke MS , Wootton JC, Lehmann MM et al Coordinated progression through two subtranscriptomes underlies the tachyzoite cycle of *Toxoplasma gondii*. PLoS One 2010;5:e12354. 20865045 10.1371/journal.pone.0012354 PMC2928733

[btae241-B7] Bynum ML , Hackebeil GA, Hart WE et al PYOMO—Optimization Modeling in Python. Springer Optimization and Its Applications, 3rd edn. Cham, Switzerland: Springer Nature, 2021.

[btae241-B8] Charikar M , Naamad Y, Rexford J et al Multi-commodity flow with in-network processing. In: Algorithmic Aspects of Cloud Computing, Lecture Notes in Computer Science. Cham: Springer International Publishing, 2019, 73–101.

[btae241-B9] Czarnewski P , Parigi SM, Sorini C et al Conserved transcriptomic profile between mouse and human colitis allows unsupervised patient stratification. Nat Commun 2019;10:2892. 31253778 10.1038/s41467-019-10769-x PMC6598981

[btae241-B10] Czarnowicki T , He H, Krueger JG et al Atopic dermatitis endotypes and implications for targeted therapeutics. J Allergy Clin Immunol 2019;143:1–11. 30612663 10.1016/j.jaci.2018.10.032

[btae241-B11] Ding J , Sharon N, Bar-Joseph Z. Temporal modelling using single-cell transcriptomics. Nat Rev Genet 2022;23:355–68. 35102309 10.1038/s41576-021-00444-7 PMC10354343

[btae241-B12] Ernst J , Bar-Joseph Z. STEM: a tool for the analysis of short time series gene expression data. BMC Bioinformatics 2006;7:191. 16597342 10.1186/1471-2105-7-191 PMC1456994

[btae241-B13] Even S , Itai A, Shamir A. On the complexity of time table and multi-commodity flow problems. In: *16th Annual Symposium on Foundations of Computer Science (SFCS 1975)*. NW Washington, DC, United States: IEEE Computer Society, 1975, 184–93.

[btae241-B14] Fang Z , Liu X, Peltz G. GSEApy: a comprehensive package for performing gene set enrichment analysis in python. Bioinformatics 2023;39:btac757. 36426870 10.1093/bioinformatics/btac757 PMC9805564

[btae241-B15] Hao Y , Stuart TIM, Kowalski MH et al Dictionary learning for integrative, multimodal and scalable single-cell analysis. Nat Biotechnol 2024;42:293–304. 37231261 10.1038/s41587-023-01767-y PMC10928517

[btae241-B16] Huang T , Cui W, Hu L et al Prediction of pharmacological and xenobiotic responses to drugs based on time course gene expression profiles. PLoS One 2009;4:e8126. 19956587 10.1371/journal.pone.0008126 PMC2780314

[btae241-B17] Johnson WE , Li C, Rabinovic A. Adjusting batch effects in microarray expression data using empirical bayes methods. Biostatistics 2006;8:118–27. 16632515 10.1093/biostatistics/kxj037

[btae241-B18] Lange M , Bergen V, Klein M et al CellRank for directed single-cell fate mapping. Nat Methods 2022;19:159–70. 35027767 10.1038/s41592-021-01346-6 PMC8828480

[btae241-B19] LaSalle TJ , Gonye ALK, Freeman SS et al Longitudinal characterization of circulating neutrophils uncovers phenotypes associated with severity in hospitalized COVID-19 patients. Cell Rep Med 2022;3:100779. 36208629 10.1016/j.xcrm.2022.100779 PMC9510054

[btae241-B20] Leighton T , Makedon F, Plotkin S et al Fast approximation algorithms for multicommodity flow problems. J Comput Syst Sci 1995;50:228–43.

[btae241-B21] Lin T-H , Kaminski N, Bar-Joseph Z. Alignment and classification of time series gene expression in clinical studies. Bioinformatics 2008;24:i147–55. 18586707 10.1093/bioinformatics/btn152 PMC2718630

[btae241-B22] Listgarten J , Neal R, Roweis S et al Multiple alignment of continuous time series. Adv Neural Inf Process Syst 2004;17:817–824.

[btae241-B23] Liu J , Chang H-W, Grewal R et al Transcriptomic profiling of plaque psoriasis and cutaneous T-cell subsets during treatment with secukinumab. JID Innov 2022;2:100094. 35757784 10.1016/j.xjidi.2021.100094 PMC9214344

[btae241-B24] Lötvall J , Akdis CA, Bacharier LB Jr, et al Asthma endotypes: a new approach to classification of disease entities within the asthma syndrome. J Allergy Clin Immunol 2011;127:355–60. 21281866 10.1016/j.jaci.2010.11.037

[btae241-B25] Ma F , Plazyo O, Billi AC et al Single cell and spatial sequencing define processes by which keratinocytes and fibroblasts amplify inflammatory responses in psoriasis. Nat Commun 2023;14:3455. 37308489 10.1038/s41467-023-39020-4 PMC10261041

[btae241-B26] Macnair W , Gupta R, Claassen M. psupertime: supervised pseudotime analysis for time-series single-cell RNA-seq data. Bioinformatics 2022;38:i290–8. 35758781 10.1093/bioinformatics/btac227 PMC9235474

[btae241-B27] Massey FJ. The kolmogorov-smirnov test for goodness of fit. J Am Stat Assoc 1951;46:68–78.

[btae241-B28] McInnes L , Healy J, Saul N et al UMAP: Uniform manifold approximation and projection. JOSS 2018;3:861.

[btae241-B29] Meindl B , Templ M. Analysis of commercial and free and open source solvers for the cell suppression problem. Trans Data Priv 2013;6:147–59.

[btae241-B30] Meyer UA , Zanger UM, Schwab M. Omics and drug response. Annu Rev Pharmacol Toxicol 2013;53:475–502. 23140244 10.1146/annurev-pharmtox-010510-100502

[btae241-B31] Oki E. Basics of linear programming. In: Linear Programming and Algorithms for Communication Networks. CRC Press, Boca Raton, FL, 2012, 19–38.

[btae241-B32] Parma V , Ohla K, Veldhuizen MG et al; GCCR Group Author. More than smell-COVID-19 is associated with severe impairment of smell, taste, and chemesthesis. Chem Senses 2020;45:609–22. 32564071 10.1093/chemse/bjaa041 PMC7337664

[btae241-B33] Povroznik JM , Robinson CM. IL-27 regulation of innate immunity and control of microbial growth. Future Sci OA 2020;6:FSO588. 32802395 10.2144/fsoa-2020-0032 PMC7421895

[btae241-B34] Robinson MD , Oshlack A. A scaling normalization method for differential expression analysis of RNA-seq data. Genome Biol 2010;11:R25. 20196867 10.1186/gb-2010-11-3-r25 PMC2864565

[btae241-B35] Saelens W , Cannoodt R, Todorov H et al A comparison of single-cell trajectory inference methods. Nat Biotechnol 2019;37:547–54. 30936559 10.1038/s41587-019-0071-9

[btae241-B36] Shibata S , Tada Y, Kanda N et al Possible roles of IL-27 in the pathogenesis of psoriasis. J Invest Dermatol 2010;130:1034–9. 19924133 10.1038/jid.2009.349

[btae241-B37] Subramanian A , Tamayo P, Mootha VK et al Gene set enrichment analysis: a knowledge-based approach for interpreting genome-wide expression profiles. Proc Natl Acad Sci USA 2005;102:15545–50. 16199517 10.1073/pnas.0506580102 PMC1239896

[btae241-B38] Traag VA , Waltman L, van Eck NJ. From Louvain to Leiden: guaranteeing well-connected communities. Sci Rep 2019;9:5233. 30914743 10.1038/s41598-019-41695-z PMC6435756

[btae241-B39] Tran TN , Bader GD. Tempora: cell trajectory inference using time-series single-cell RNA sequencing data. PLoS Comput Biol 2020;16:e1008205. 32903255 10.1371/journal.pcbi.1008205 PMC7505465

[btae241-B40] VanDussen KL , Stojmirović A, Li K et al Abnormal small intestinal epithelial microvilli in patients with Crohn’s disease. Gastroenterology 2018;155:815–28. 29782846 10.1053/j.gastro.2018.05.028 PMC6378688

[btae241-B41] Wagner MFMG , Theodoro TR, Filho CDASM et al Extracellular matrix alterations in the skin of patients affected by psoriasis. BMC Mol. Cell Biol 2021;22:55. 34715781 10.1186/s12860-021-00395-1 PMC8555298

[btae241-B42] Wang Y , Mashock M, Tong Z et al Changing technologies of RNA sequencing and their applications in clinical oncology. Front Oncol 2020;10:447. 32328458 10.3389/fonc.2020.00447 PMC7160325

