# Supplementary material for: Integrating patients in time series clinical transcriptomics data
Source: Bioinformatics. 2024 Jun 28;40(Suppl 1):i151–9. doi: 10.1093/bioinformatics/btae241 (PMC11256926; doi:10.1093/bioinformatics/btae241)
Supplement: btae241_Supplementary_Data [file btae241_supplementary_data.pdf]

Supplement: Integrating patients in time series clinical transcriptomics data

Euxhen Hasanaj[1], Sachin Mathur[2], and Ziv Bar-Joseph[1,2,3]

[1]Machine Learning Department, School of Computer Science, Carnegie Mellon University, Pittsburgh, PA, 15213, USA
[2]R&D Data and Computational Sciences, Sanofi, Cambridge, MA, 02141, USA
[3]Computational Biology Department, School of Computer Science, Carnegie Mellon University, Pittsburgh, PA, 15213, USA

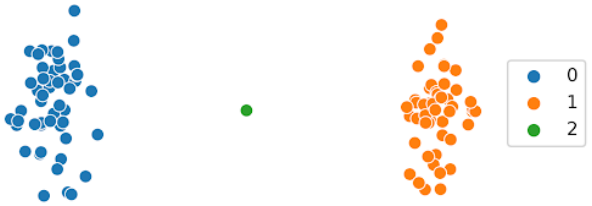

Fig. 1: **Toy example demonstrating the need for node capacities.** Assume a situation where we have a single outlier (in green). Without capacities, we would incorrectly identify the trajectory 0-2-1 since all patients would travel through the green node. With node capacities, however, only one patient is allowed to travel through 2, and the rest travel through the more likely disease path 0-1. The possibility of such outliers impacting the results depends on the size of the dataset and the sampling rates.

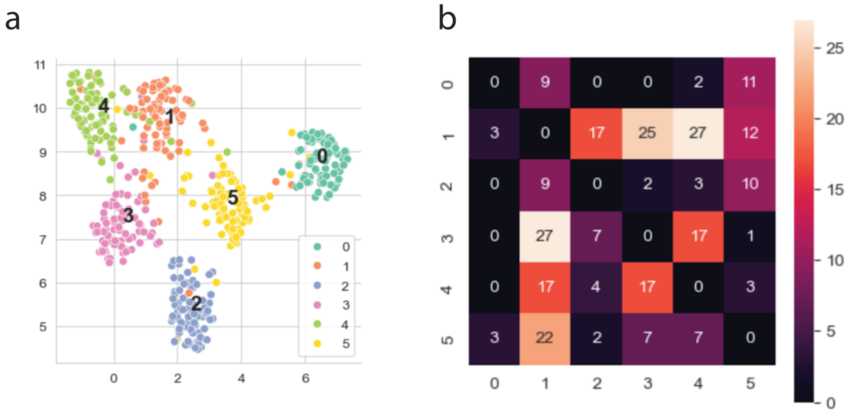

Fig. 2: **Truffle on simulated data.** (a) We randomly generated 15-dimensional samples from 6 different states and constructed random patient trajectories from these (by randomly deleting some nodes to simulate sparse visits). We designated 0-5-1-4, 5-1-3-4, and 4-3-1-2-5 as "true trajectories". From these, Truffle accurately identified 4-3-1-2-5 as the top trajectory of length 4, and also 5-1-3-4 as the top trajectory of length 3. The other path 0-5-1-4 was one of the top three trajectories of length 3.

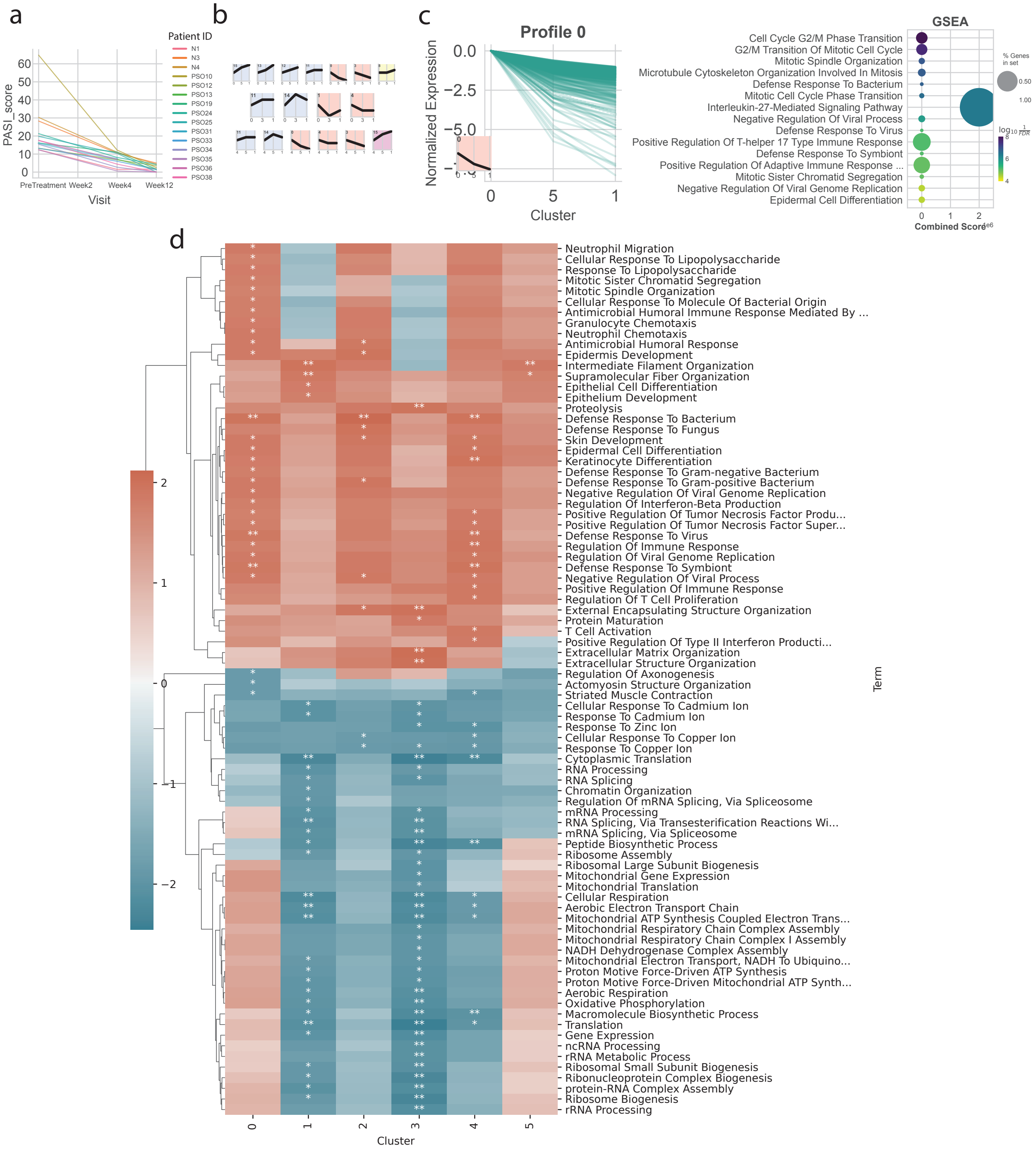

Fig. 3: **Supplementary plots for the psoriasis dataset.** (a) PASI scores for each patient. (b) All significant STEM profiles for all three trajectories identified by Truffle. (c) Profile 0 for the trajectory $0 - 5 - 1$ shows a high score for "IL-27-Mediated Signaling Pathway." (d) Complete heatmap with all significant GO processes (FDR < 0.05).

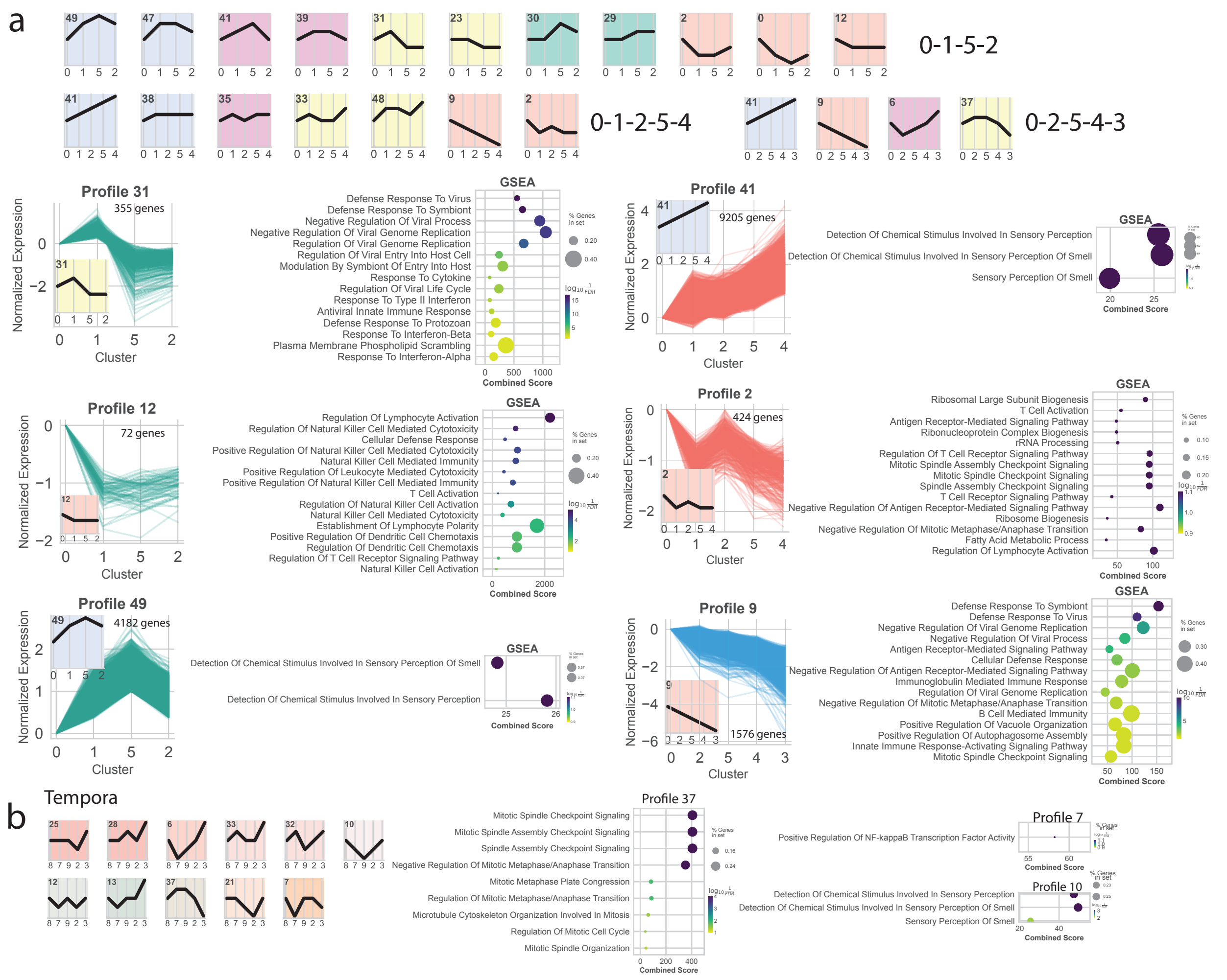

Fig. 4: **Supplementary plots for the COVID-19 dataset.** (a) Selected STEM profiles and their GO processes. (b) STEM profiles for Tempora's trajectory $8-7-9-2-3$ along with GO processes for profiles 37, 7, and 10.

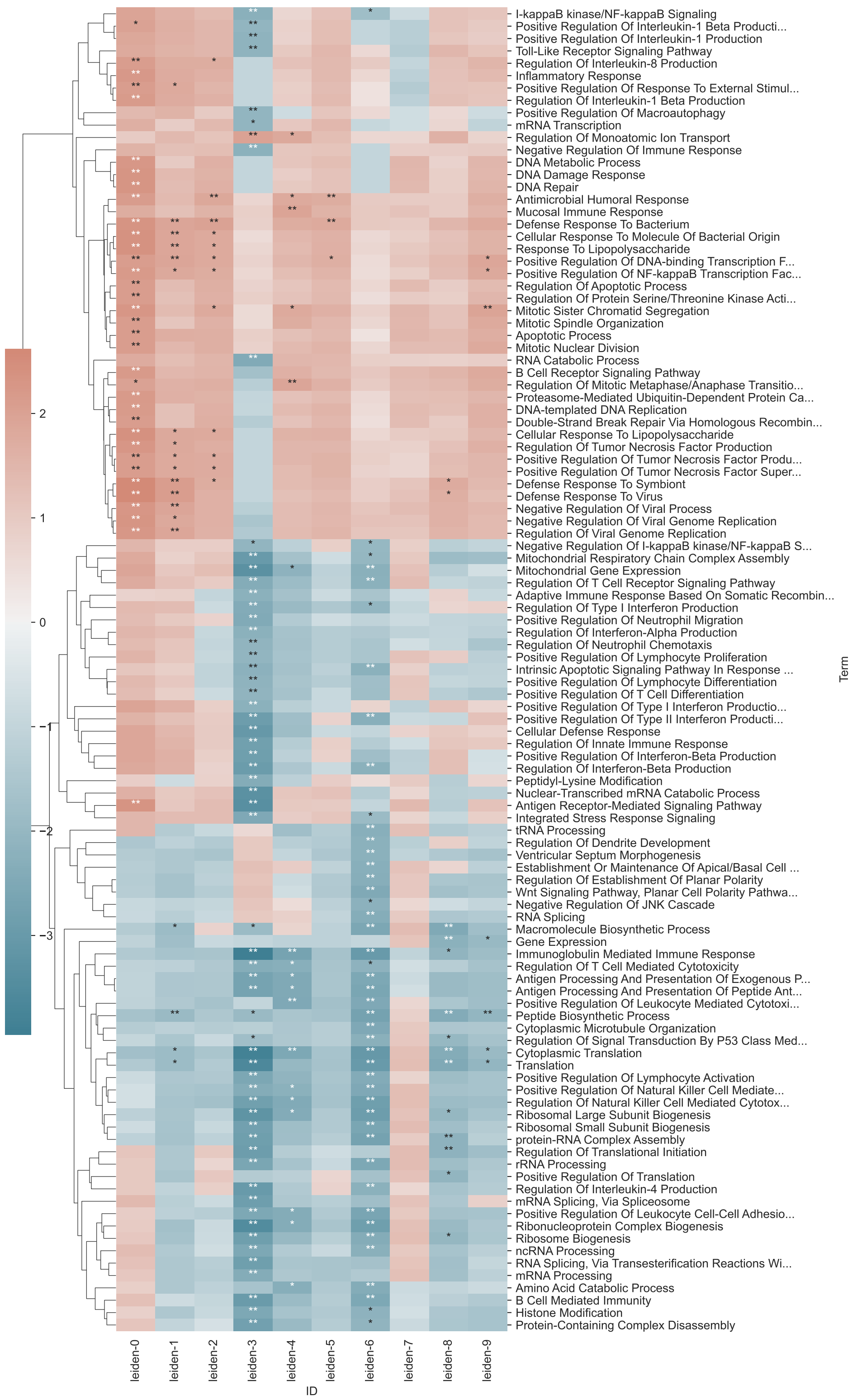

Fig. 5: GO processes for each cluster in the COVID-19 data.

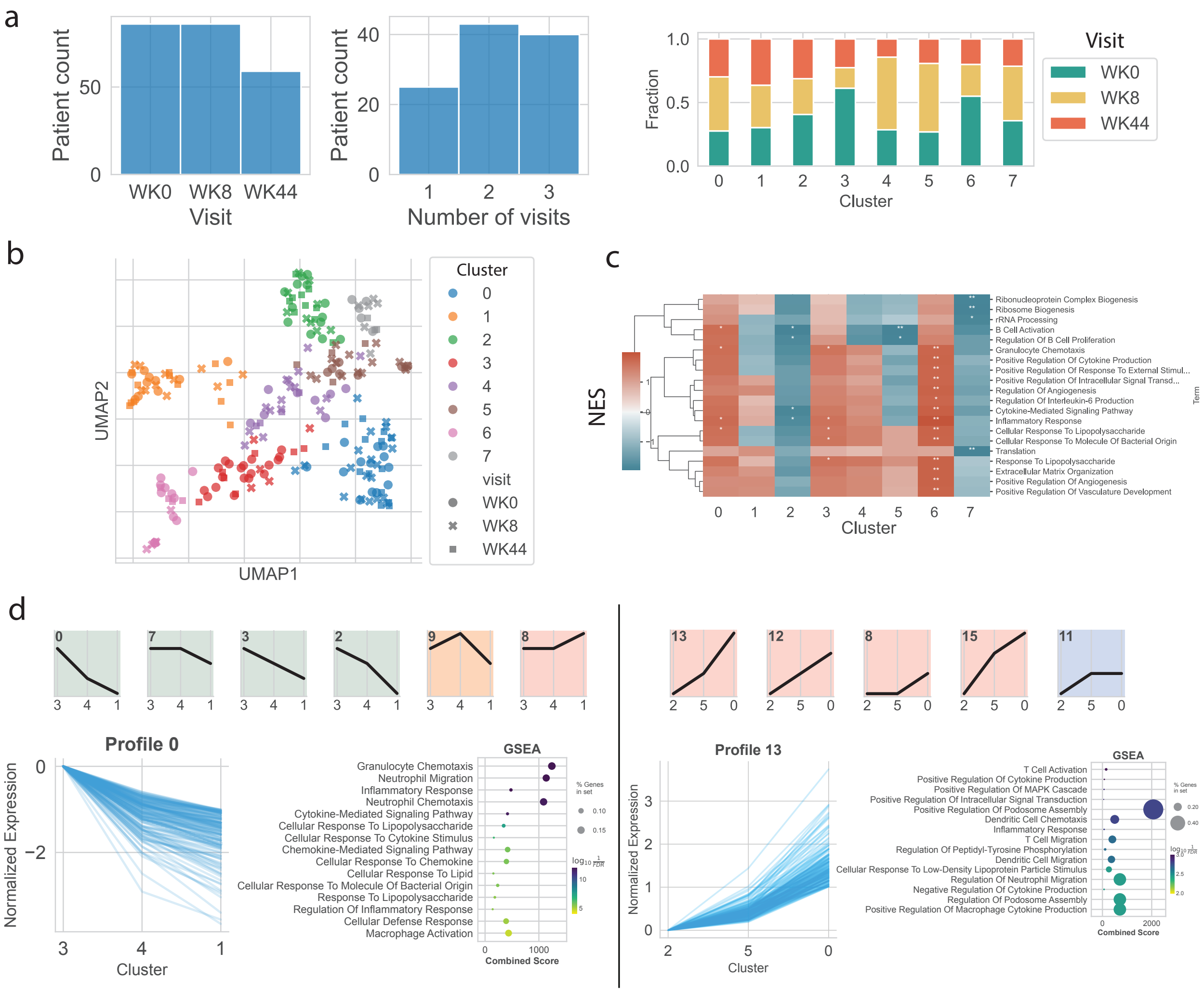

Fig. 6: Supplementary plots for the Crohn's disease dataset. (a) Information on the number of visits per patient and cluster. (b) Clustering results. (c) Top GO processes for each cluster when compared to healthy samples. (d) STEM profiles for the top two paths $3-4-1$ and $2-5-0$ along with GO processes for the top profile for each.
